# Synthesis and Antimicrobial Activity of Novel Ag-*N*-Hetero-cyclic Carbene Complexes

**DOI:** 10.3390/molecules15042499

**Published:** 2010-04-08

**Authors:** İlknur Özdemir, Emine Özge Özcan, Selami Günal, Nevin Gürbüz

**Affiliations:** 1 Department of Chemistry, Faculty of Science and Art, İnönü University, 44280 Malatya, Turkey; E-Mail: eozgeozcan@gmail.com (E.O.O.); 2 Department of Microbiology, Faculty of Medicine, İnönü University, 44280 Malatya, Turkey; E-Mail: selamig@inonu.edu.tr (S.G.)

**Keywords:** *N*-Heterocyclic carbene, silver complexes, antimicrobial activity, imidazolidin-2-ylidene

## Abstract

A series of imidazolidinium ligand precursors are metallated with Ag_2_O to give silver(I) *N*-heterocyclic carbene complexes. All compounds were fully characterized by elemental analyses, ^1^H-NMR, ^13^C-NMR and IR spectroscopy techniques. All compounds studied in this work were screened for their *in vitro* antimicrobial activities against the standard strains: *Enterococcus faecalis (ATCC 29212), Staphylococcus aureus (ATCC 29213), Escherichia coli (ATCC 25922), Pseudomonas aeruginosa (ATCC 27853) *and the fungi *Candida albicans* and *Candida tropicalis*. The new imidazolidin-2-ylidene silver complexes have been found to display effective antimicrobial activity against a series of bacteria and fungi.

## 1. Introduction

With metallopharmaceuticals playing a significant role in therapeutic and diagnostic medicine, the discovery and development of new metallodrugs remain an ever-growing area of research in medicinal inorganic chemistry [1,2,3]. Metallic silver, silver salts and silver complexes have been used in a variety of applications like water purification, wound management, eye-drops, anti-infective coatings in medical devices and in burn treatment because they have potent antimicrobial properties but with low human toxicity [4,5,6,7,8,9]. Among the various silver containing species, silver complexes are particularly interesting since the antimicrobial activity can be changed by varying type of ligands coordinated to silver. The Ag(I) imidazolate complex has antibacterial and antifungal properties [10]. The phosphine adduct of Ag(I) imidazolate has essentially no antimicrobial activity [11]. In addition, an anticancer activity of silver and silver complexes has been demonstrated recently [12,13]. 

The biomedical applications of metal complexes based on *N*-heterocyclic carbine (NHC) [14,15,16,17,18] are just beginning to unfold, despite such complexes being phenomenally successful in homogeneous catalysis [19,20,21]. *N*-Heterocyclic carbene complexes of Ag are commonplace in the organometallic literature. The interest in Ag-NHC complexes is largely due to their ease of synthesis and their ability to serve as useful to other NHC-metal complexes by NHC transfer reactions [22].In addition, their diverse properties in bonding and structure and potential applications in medicine [23,24,25,26,27,28], nanomaterials [29], liquid crystals [30] and organic catalysis [31] also contribute to the attraction of Ag-NHCs. 

Ag-carbene complexes derived from imidazolium salts were synthesized and characterized for the first time by Arduengo in 1993 [32]. These complexes were obtained by reaction of the free carbene with silver triflate. Bertrand and co-workers first used silver acetate as a silver base to react triazolium salts to synthesize polymeric Ag-NHC complexes [33]. The use of silver oxide to give silver complexes of 1,3-diethylbenzimidazole-2-ylidine was pioneered by Lin and co-worker [34]. More recently, Danopoulos and co-workers reported the use of silvercarbonate to deprotonate imidazolium salts to give silver-NHC complexes [35]. 

The finding silver based antimicrobials active against bacteria, we report the preparation and characterization of the imidazolidin-2-ylidene silver(I) (**1**) isolated high yield, by complete elemental analyses, FT-IR, ^1^H and ^13^C-NMR spectroscopy. 

## 2. Results and Discussion

### 2.1. Preparation of silver-carbene complexes 1a-f

Three common approaches towards the synthesis of Ag–NHC complexes are: i) the reaction of a free NHC with silver salts [32], ii) the reaction of azolium salts with silver salts under basic phase-transfer conditions [36,37] and iii) the reaction of azolium salts with silver bases [30,38,39]. The latter method, in which Ag_2_O is used as a base is now by far the most commonly employed [22]. Soon after the first report in 199 [34], the Ag_2_O route was recognized for its attractive features, such as its stability towards air and the tolerance towards other reactive hydrogen atoms. This method was used for the preparation of complexes **1a**-**1f**. 

**Scheme 1:**
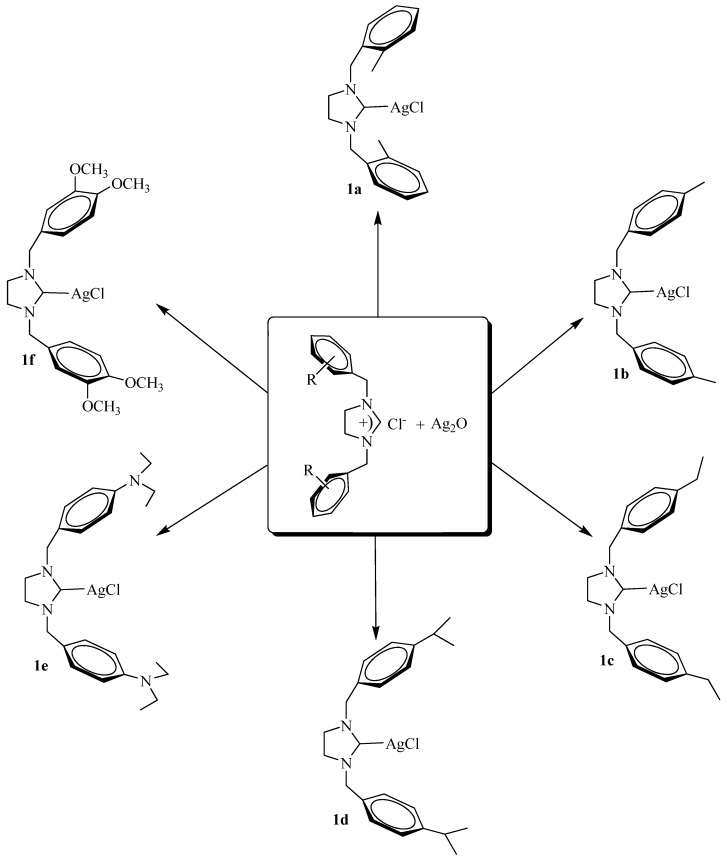
Synthesis of silver-carbene complexes.

The ligand precursor 1,3-dialkylimidazolidinium chloride was obtained according to literature [40]. Treatment of the imidazolidinium salts with 0.5 equiv. of Ag_2_O in CH_2_Cl_2_ afforded quantitavely after 24 hours the expected carbenes **1a**-**1f** (Scheme 1). Silver-carbene complexes **1a‑f** were obtained as white solids in 91–95% yields. The silver carbene complexes (**1a**–**f**) were soluble in halogenated solvents and insoluble in non-polar solvents.

Their ^1^H and ^13^C-NMR spectra are consistent with the proposed formula. In the ^1^H-NMR and ^13^C-NMR spectra of this solid product in DMSO-d_6_ and CDCl_3_ loss of the imidazolidinium proton (NC*H*N) and imidazolidinium carbon (N*C*HN) signal suggests the formation of the silver-NHC complexes. The ^13^C-NMR spectra exhibit singlets at 203.1, 202.1 and 202.9 ppm for **1b**, **1c** and **1f** respectively characteristic of the carbenic carbon resonance. The chemical shift is well consistent with those of the knows silver-NHC complexes in the range of 213-163 ppm [41,42]. In the other complexes, the resonances for carbene carbons were not detected, which was also mentioned in the literature and given a reason of the fluxional behavior of the NHCs complexes [43,44,45]. Ag-NHC complexes exhibit a characteristic υ_(NCN)_ band typically at 1,501, 1,500, 1,515, 1,502, 1,507 and 1,508 cm^-1^,respectively, for **1a-f**.

### 2.2. Antimicrobial properties of silver-NHC complexes

The antimicrobial activity was reported in terms of the minimum inhibitory concentration (MIC) values, which are defined as the lowest concentration of an antimicrobial that visibly inhibits the growth of the bacteria after an overnight incubation [46]. The usefulness of **1a**-**f** as antimicrobial agents was evaluated. Minimal inhibitory concentrations for each compound were investigated against standard bacterial strains; *Staphylococcus aureus* ATCC 29213, *Enterococcus faecalis* ATCC 29212, *Escherichia coli* ATCC 25922, *Pseudomonas aeruginosa* ATCC 27853 and the fungal strains *Candida albicans* and *Candida tropicalis.* The test organism was laboratory strains used to test a range of concentration of the silver compounds for minimum inhibitory concentration determination. Antimicrobial activities of the Ag (I)-NHC complexes were determined by using agar dilution procedure and were tested with different concentrations of the compounds. The minimum inhibitory concentration (MIC) of synthesized silver complexes against Gram positive, Gram negative bacteria and fungus are summarized in Table 1. Ampicillin, ciprofloxacin and fluconazole were used as standard drugs for comparison. 

**Table 1:** Minimum inhibitory concentration (μg/mL) of silver NHC complexes tested against bacterial and fungus.

Ag-NHC	*E. coli*	*S. aureus*	*E. faecalis*	*P. aerug.*	*C. albicans*	*C. tropicalls*
**1a**	100	100	100	100	50	12.5
**1b**	200	200	200	200	100	50
**1c**	200	200	200	200	6.25	6.25
**1d**	200	100	100	200	100	100
**1e**	100	100	100	100	25	25
**1f**	100	100	100	100	50	6.25
**Ampicillin**	3.12	3.12	1.56	-	-	-
**Ciprofloxacin**	1.56	0.39	0.78	3.12	-	-
**Fluconazole**	-	-	-	-	3.12	3.12

As shown in the table, antimicrobial activity against bacteria and fungi was observed in the silver-NHC complexes tested at 200–6.25 μg/mL concentrations. The new complexes showed effective activities against Gram-positive, Gram-negative bacteria and fungi. The complexes were found effective in inhibiting the growth of Gram-positive and Gram-negative bacteria with MICs values between 100–200 µg/mL. The tested compounds showed antifungal activity with a range of the MICs between 6.25 and 100 μg/mL. However, among the silver complexes tested **1a**, **1c** and **1f** shoved high activity against the fungi *C. albians* and *C. tropicalls *with a range of MICs between 6.25–50 µg/mL. Incorporation of same groups at positions 2 and 3 on aromatic group (**1a** and **1b**) exhibit difference in activity (**1a** is more active than **1b**). In case of incorporation of an *i*-propyl group at position 4 on aromatic ring (**1d**) decreases the activity. The ethyl group on aromatic ring (**1c**) particularly enhanced the antifungal activity. From the data obtained in this work, it is suggested that the substituents on the *N*-atom may play a crucial role in the antimicrobial activity.

## 3. Experimental

### 3.1. General

All reactions for the preparation of imidazolidinium salts and silver (NHC) complexes were carried out under argon in flame-dried glassware using standard Schlenk techniques. The solvents used were purified by distillation over the drying agents indicated and were transferred under Ar: Et_2_O (Na/K alloy), CH_2_Cl_2_ (P_4_O_10_), hexane, toluene (Na). Melting points were determined in glass capillaries under air with an Electrothermal-9200 melting point apparatus. FTIR spectra were recorded as KBr pellets in the range 400–4000 cm^–1^ with an ATI UNICAM 1000 spectrometer. ^1^H-NMR and ^13^C-NMR spectra were recorded with a Varian AS 400 Merkur spectrometer operating at 400 MHz (^1^H), 100 MHz (^13^C) in CDCl_3_ and DMSO-d_6_ with tetramethylsilane as an internal reference. Elemental analyses were performed by Turkish Research Council (Ankara, Turkey) Microlab. 

### 3.2. General method for the preparation of silver NHC complexes

A solution of imidazolidinium salt (1.0 mmol), Ag_2_O (0.5 mmol) and activated 4Ǻ molecular sieves in dichloromethane (30 mL) was stirred room temperature for 24 hours. The reaction mixture was filtered through celite and the solvent removed under reduced pressure. The crude product was recrystallized from dichloromethane/hexane at room temperature.

*Chloro-1,3-bis(2-methylbenzyl)imidazolidin-2-ylidenesilver (I) *(**1a**). Yield: 0.20 g; 90%, m.p.: 155–156 °C; υ_(CN)_ = 1,501 cm ^-1^. ^1^H-NMR (CDCl_3_) δ: 2.37 (s, 6H, CH_2_C_6_H_4_(C*H*_3_)-2), 3.44 (s, 4H, NC*H*_2_C*H*_2_N), 4.78 (s, 4H, C*H*_2_C_6_H_4_(CH_3_)-2), 7.11–7.29 (m, 8H, CH_2_C_6_*H*_4_(CH_3_)-2). ^13^C{H}-NMR (CDCl_3_) δ: 19.2 (CH_2_C_6_H_2_(*C*H_3_)-2), 48.6 (N*C*H_2_*C*H_2_N), 53.5 (*C*H_2_C_6_H_4_(CH_3_)-2), 127.0, 128.4, 128.5, 131.0, 136.7 ve 138.1 (CH_2_*C*_6_H_4_(CH_3_)-2). Anal. Calcd for C_19_H_23_AgClN_2_: C, 53.98; H, 5.48; N, 6.63%; found: C, 53.90; H, 5.43; N, 6.59. 

*Chloro-1,3-bis(4-methylbenzyl)imidazolidin-2-ylidenesilver (I) *(**1b**). Yield: 0.21 g; 95%, m.p.: 230–231 °C; υ_(CN)_ = 1,500 cm ^-1^. ^1^H-NMR (DMSO-d_6_) δ: 2.30 (s, 6H, CH_2_C_6_H_4_(C*H*_3_)-4), 3.49 (s, 4H, NC*H*_2_C*H*_2_N), 4.66 (s, 4H, C*H*_2_C_6_H_4_(CH_3_)-4), 7.09–7.24 (m, 8H, CH_2_C_6_*H*_4_(CH_3_)-4). ^13^C{H}-NMR (DMSO-d_6_) δ: 21.2 (CH_2_C_6_H_2_(*C*H_3_)-4), 48.9 (N*C*H_2_*C*H_2_N), 54.2 (*C*H_2_C_6_H_4_(CH_3_)-4), 128.2, 129.8, 133.6, 137.6 (CH_2_*C*_6_H_4_(CH_3_)-4), 203.1 (*C_carb_*)_. _Anal. Calcd for C_19_H_23_AgClN_2_: C, 53.98; H, 5.48; N, 6.63%; found: C, 53.96; H, 5.40; N, 6.62%. 

*Chloro-1,3-bis(4-ethylbenzyl)imidazolidin-2-ylidenesilver (I)* (**1c**). Yield: 0.20 g; 92%, m.p.: 97–98 °C; υ_(CN)_ = 1,515 cm ^-1^. ^1^H-NMR (DMSO-d_6_) δ: 1.12 (t, *J* = 7.5 Hz, 6H, CH_2_C_6_H_4_(CH_2_C*H*_3_)-4), 2.54 (q, *J* = 7.5 Hz, 4H, CH_2_C_6_H_4_(C*H*_2_CH_3_)-4), 3.46 (s, 4H, NC*H*_2_C*H*_2_N), 4.52 (s, 4H, C*H*_2_C_6_H_4_(CH_2_CH_3_)-4), 7.07 ve 7.22 (d, *J *= 7.8 Hz, 8H, CH_2_C_6_*H*_4_(CH_2_CH_3_)-4). ^13^C{H}-NMR (DMSO-d_6_) δ: 14.6 (CH_2_C_6_H_2_(CH_2_*C*H_3_)-4), 27.3 (CH_2_C_6_H_2_(*C*H_2_CH_3_)-4), 46.3 (N*C*H_2_*C*H_2_N), 50.5 (*C*H_2_C_6_H_4_(CH_2_CH_3_)-4), 127.3, 128.2, 128.7, 143.2 (CH_2_*C*_6_H_4_(CH_2_CH_3_)-4), 202.1 (*C_carb_*)_. _Anal. Calcd for C_21_H_27_AgClN_2_: C, 55.95; H, 6.04; 7.86; N, 6.21%; found: C, 55.90; H, 5.98; N, 6.26%. 

*Chloro-1,3-bis(4-i-propylbenzyl)imidazolidin-2-ylidenesilver (I) * (**1d**). Yield: 0.20 g; 94%, m.p.: 194–195 °C; υ_(CN)_ = 1,503 cm ^-1^. ^1^H-NMR (DMSO-d_6_) δ: 1.88 (d, *J* = 6.9 Hz, 12H, CH_2_C_6_H_4_(CH(C*H*_3_)_2_)-4), 2.87 (h, *J* = 6.9 Hz, 2H, CH_2_C_6_H_4_(C*H*(CH_3_)_2_)-4), 3.56 (s, 4H, NC*H*_2_C*H*_2_N), 4.6 (s, 4H, C*H*_2_C_6_H_4_(CH(CH_3_)_2_)-4), 7.18 ve 7.29 (d, *J* = 5.1 Hz, 8H, CH_2_C_6_*H*_4_(CH(CH_3_)_2_)-4). ^13^C{H}-NMR (DMSO-d_6_) δ: 22.6 (CH_2_C_6_H_2_(CH(*C*H_3_)_2_)-4), 33.4 (CH_2_C_6_H_2_(*C*H(CH_3_)_2_)-4), 46.6 (N*C*H_2_*C*H_2_N), 53.0 (*C*H_2_C_6_H_4_(CH(CH_3_)_2_)-4), 126.2, 127.9, 129.8, 148.9 (CH_2_*C*_6_H_4_(CH_3_)-4). Anal. Calcd for C_23_H_31_AgClN_2_: C, 57.69; H, 6.53; N, 5.85%; found: C, 57.63; H, 6.50; N, 5.83%.

*Chloro-1,3-bis(4-diethylaminobenzyl)imidazolidin-2-ylidenesilver (I) * (**1e**). Yield: 0.19g; 91%, m.p.: 163–165 °C; υ_(CN)_ = 1,507 cm ^-1^. ^1^H-NMR (CDCl_3_) δ: 1.15 (t, *J* = 7.05 Hz, 12H, CH_2_C_6_H_4_N(CH_2_C*H*_3_)_2_-4), 3.36 (q, *J = *6.9 Hz, 8H, CH_2_C_6_H_4_N(C*H*_2_CH_3_)_2_-4), 3.69 (s, 4H, NC*H*_2_C*H*_2_N), 4.57 (s, 4H, C*H*_2_C_6_H_4_N(CH_2_CH_3_)_2_-4), 6.61 ve 6.64 (d, *J = *9.0 Hz, 8H, CH_2_C_6_*H*_4_N(CH_2_CH_3_)_2_-4). ^13^C{H}-NMR (CDCl_3_) δ: 12.5 (CH_2_C_6_H_2_N(CH_2_*C*H_3_)_2_-4), 44.3 (CH_2_C_6_H_2_N(*C*H_2_CH_3_)_2_-4), 48.2 (N*C*H_2_*C*H_2_N), 52.0 (*C*H_2_C_6_H_4_N(CH_2_CH_3_)_2_-4), 111.7, 121.1, 130.3, 147.7 (CH_2_*C*_6_H_4_N(CH_2_CH_3_)_2_-4). Anal. Calcd for C_25_H_37_AgClN_4_: C, 55.93; H, 6.95; N, 10.44%; found: C, 55.90; H, 6.93; 6.63; N, 10.42%. 

*Chloro-1,3-bis(3,4-dimethoxybenzyl)imidazolidin-2-ylidenesilver (I) *(**1f**). Yield: 0.19 g; 93%, m.p.:134–135 °C; υ_(CN)_ = 1,508 cm ^-1^. ^1^H-NMR (DMSO-d_6_) δ: 3.36 (s, 4H, NC*H*_2_C*H*_2_N), 3.53 (s, 6H, CH_2_C_6_H_3_(OC*H*_3_)_2_-3), 3.75 (s, 6H, CH_2_C_6_H_3_(OC*H*_3_)_2_-4), 4.63 (s, 4H, C*H*_2_C_6_H_3_(OCH_3_)_2_-3,4), 6.76-7.01(m, 6H, CH_2_C_6_*H*_3_(OCH_3_)_2_-3,4). ^13^C{H}-NMR (DMSO-d_6_) δ: 49.0 (N*C*H_2_*C*H_2_N), 54.2 (CH_2_C_6_H_3_(O*C*H_3_)_2_-3), 55.9 (CH_2_C_6_H_3_(O*C*H_3_)_2_-4), 56.0 (*C*H_2_C_6_H_3_(OCH_3_)_2_-3,4), 112.1, 112.4, 120.4, 129.0, 148.9, 149.3 (CH_2_*C*_6_H_3_(OCH_3_)_2_-3,4), 202.9 (*C_carb_*)_. _Anal. Calcd for C_21_H_27_AgClN_2_O_4_: C, 49.00; H, 5.29; N, 5.44; O, 12.43%; found: C, 49.02; H, 5.30; N, 5.40; O, 12.44%. 

### 3.3. Antimicrobial activities of silver NHC complexes

Antimicrobial activities of the Ag (I) complexes with *N*-Heterocyclic carbene ligand were determined using the agar dilution procedure recommended by the Clinical and Laboratory Standards Institute [47,48]. Minimal inhibitory concentrations for each compound were investigated against standard bacterial strains; *Staphylococcus aureus* ATCC 29213, *Enterococcus faecalis* ATCC 29212, *Escherichia coli* ATCC 25922, *Pseudomonas aeruginosa* ATCC 27853 were obtained from American Type Culture Collection (Rockville, MD.) and the fungal strains *Candida albicans* and *Candida tropicalis* obtained from the Department of Microbiology, Faculty of Medicine, Ege University (Turkey). Bacterial strains were subcultured on Muller Hinton Broth (HiMedia Laboratories Pvt. Ltd. Mumbai-India) and fungal strains were also on RPMI 1640 Broth (Sigma-Aldrich Chemie GmbH Taufkirchen, Germany). Their turbidities matched that of a McFarland no. 0.5 turbidity standard [49]. The stock solution of all compounds was prepared in dimethyl sulfoxide (DMSO). All of the dilutions were done with distilled water. The concentrations of the tested compounds were 800, 400, 200, 100, 50, 25, 12.5 and 6.25 µg/mL. Ampicillin and ciprofloxacin were used as antibacterial standard drugs, while fluconazole were used as antifungal standard drugs whose minimum inhibitory concentration (MIC) values are provided. A loopful (0.01 mL) of the standardised inoculums of the bacteria and yeasts (10^6^ CFUs/mL) was spread over the surface of agar plates. All the inoculated plates were incubated at 35 °C and results were evaluated after 16–20 h of incubation for bacteria and 48 h for yeasts. The lowest concentration of the compounds that prevented visible growth was considered to be the minimal inhibitory concentration (MIC).

## 4. Conclusions

In summary, six Ag(I)-NHC complexes were synthesized starting from 1,3-dialkylimidazolidinium chloride, following a common procedure. The silver-NHC complexes characterized by ^1^H-NMR, ^13^ C- NMR, IR and elemental analysis. Also, antimicrobial activities of the new complexes are reported. The silver complexes **1a**, **1c** and **1f** showed better antimicrobial activity against the fungi than other complexes, even at the much lower concentrations. Although the mechanism of antimicrobial activity is not known, it was found that substituents on the *N*-atom have an effect on antimicrobial activity in this work. Detailed investigations focusing on new silver-NHC complexes and other biomedical applications are ongoing. 
